# Plasma IAPP-Autoantibody Levels in Alzheimer’s Disease Patients Are Affected by *APOE4* Status

**DOI:** 10.3390/ijms24043776

**Published:** 2023-02-14

**Authors:** Dovilė Pocevičiūtė, Bodil Roth, Nina Schultz, Cristina Nuñez-Diaz, Shorena Janelidze, The Netherlands Brain Bank, Anders Olofsson, Oskar Hansson, Malin Wennström

**Affiliations:** 1 Cognitive Disorder Research Unit, Department of Clinical Sciences Malmö, Lund University, 214 28 Malmö, Sweden; 2 Department of Internal Medicine, Lund University, Skåne University Hospital, 214 28 Malmö, Sweden; 3 Clinical Memory Research Unit, Department of Clinical Sciences Malmö, Lund University, 223 62 Lund, Sweden; 4 Netherlands Institute for Neuroscience, 1105 BA Amsterdam, The Netherlands; 5 Department of Medical Biochemistry and Biophysics, Umeå University, 901 87 Umeå, Sweden; 6 Memory Clinic, Skåne University Hospital, 212 24 Malmö, Sweden

**Keywords:** AD, amylin, amyloid beta, *APOE4*, autoantibodies, cognition, IgA, IgG, IgM, T2D

## Abstract

Pancreas-derived islet amyloid polypeptide (IAPP) crosses the blood–brain barrier and co-deposits with amyloid beta (Aβ) in brains of type 2 diabetes (T2D) and Alzheimer’s disease (AD) patients. Depositions might be related to the circulating IAPP levels, but it warrants further investigation. Autoantibodies recognizing toxic IAPP oligomers (IAPP_O_) but not monomers (IAPP_M_) or fibrils have been found in T2D, but studies on AD are lacking. In this study, we have analyzed plasma from two cohorts and found that levels of neither immunoglobulin (Ig) M, nor IgG or IgA against IAPP_M_ or IAPP_O_ were altered in AD patients compared with controls. However, our results show significantly lower IAPP_O_-IgA levels in apolipoprotein E (*APOE) 4* carriers compared with non-carriers in an allele dose-dependent manner, and the decrease is linked to the AD pathology. Furthermore, plasma IAPP-Ig levels, especially IAPP-IgA, correlated with cognitive decline, C-reactive protein, cerebrospinal fluid Aβ and tau, neurofibrillary tangles, and brain IAPP exclusively in *APOE4* non-carriers. We speculate that the reduction in IAPP_O_-IgA levels may be caused by increased plasma IAPP_O_ levels or masked epitopes in *APOE4* carriers and propose that IgA and *APOE4* status play a specific role in clearance of circulatory IAPP_O_, which may influence the amount of IAPP deposition in the AD brain.

## 1. Introduction

Alzheimer’s disease (AD) is a heterogenous disorder characterized by an accumulation of extracellular amyloid beta (Aβ) plaques and hyperphosphorylated tau (p-tau), forming the so-called intraneuronal fibrillary tangles (NFT) [1,2]. The disease occurs in familial and sporadic forms, where the latter accounts for more than 90% of the disease cases [3]. The highest risk factor of sporadic AD is age, but carrying certain gene isoforms has also been shown to significantly increase the risk of AD [4,5]. The most studied AD risk gene is apolipoprotein E (*APOE*), whose encoded protein APOE mediates the binding of lipoproteins or lipid complexes in the plasma or interstitial fluids to specific cell-surface receptors [6]. There are three main isoforms: *APOE2*, *APOE3*, and *APOE4*. The *APOE2* is the least common isoform, whereas *APOE3* is the most common, carried by 8% and 77% of the population, respectively [7]. While *APOE2* appears to be protective against sporadic AD [8], the *APOE4* variant is strongly associated with an increased risk of AD [7], and the age of AD onset decreases with the number of *APOE4* alleles [9]. In what way *APOE4* contributes to AD pathology is still under investigation, but experimental studies have demonstrated that APOE4 exacerbates the Aβ plaque and tau burden and disrupts glial immunomodulating functions leading to chronic inflammation [10].

Sporadic AD is also associated with disorders linked to vascular dysfunction. In particular, type 2 diabetes (T2D) has been put forward, as the risk of developing AD is 1.5-fold higher in this patient group [11]. The risk is strongly linked to the vascular complications associated with the disease [12], but previous studies have also highlighted a potential implication of islet amyloid polypeptide (IAPP, also called amylin, insulinoma amyloid peptide, or diabetes-associated peptide). This 37-amino acid-long pancreas-derived peptide is known to form toxic deposits in peripheral organs (e.g., pancreas, kidney, and heart) in T2D patients [13], but studies have shown that it also forms deposits in the brain, often in vessel walls [14,15]. These depositions are linked to vessel wall disruption in both T2D patients and rats overexpressing human IAPP [16]. Since IAPP has also been found in AD hippocampal tissue as inclusions within vessel-supporting pericytes showing apoptotic features [17], it is tempting to speculate that IAPP accumulation plays a role in the increased risk of AD in T2D patients with vascular complications. Indeed, evidence points towards a link between AD pathology and IAPP. Immunohistological stainings show that IAPP often co-localizes with Aβ42 in plaques and Aβ40 in vessel walls [14,15]. Furthermore, a recent study using human and animal models has demonstrated that pancreas-derived IAPP accumulates in circulating monocytes and co-deposits with Aβ within the brain microvasculature, further inducing cerebrovascular inflammation [18]. IAPP (and its non-amyloidogenic analogue pramlintide) also plays a crucial role in Aβ brain-to-blood clearance [18,19,20]. Interestingly, this clearance, especially of Aβ40, is attenuated in the presence of *APOE4* [21], leading to an exacerbated IAPP deposition and vascular pathology [16]. APOE has also been shown to interfere with IAPP aggregation in an allele-dependent way and protect pericytes from IAPP-induced toxicity, with the APOE4 variant being the least protective [22]. 

To investigate the potential link between IAPP and AD pathology further, we have, in a previous study, measured plasma IAPP levels in AD patients and healthy controls [23]. Although we found an inverse correlation between plasma IAPP levels and cerebrospinal fluid (CSF) Aβ levels in AD patients (indicative of Aβ accumulation in the brain) [23], we did not detect any significant differences in plasma IAPP levels between AD patients and healthy controls. Our plasma samples were, however, not fasting samples; hence, the individual and circadian fluctuations of IAPP could have influenced the results. Within the T2D research field, this problem has been worked around by measuring endogenous IAPP-autoantibody levels instead, since these could reflect the IAPP levels without the influence of the circadian fluctuations. This idea is partly based on a previous mouse study demonstrating increased levels of immunoglobulin (Ig) G against aggregated but not soluble IAPP after injection with a vaccine containing IAPP peptides [24]. Furthermore, higher blood levels of IAPP-autoantibodies have been found in T2D patients compared with non-diabetic subjects [25], and specific autoantibodies directed against IAPP oligomers (but not monomers or fibrils) have been exclusively found in diabetic patients [26], confirming the pathological relevance of the amyloidogenic peptide in T2D.

Autoantibodies are self-reactive antibodies found in the blood, colostrum, saliva, and CSF of all mammals, regardless of age, sex, or the presence of disease. The most prominent Ig isotypes are IgM, IgG, and IgA, where IgM is produced by B cells in the primary immune response. B cells subsequently differentiate into other types of B cells which produce IgG and, in a smaller amount, IgA. The former is one of the most abundant proteins in human blood, produced in a delayed response to an infection, while the latter, found monomeric in serum and dimeric in mucosa (e.g., saliva, tears, colostrum, intestinal and genital tract, and respiratory secretions), far exceeds the combined total amounts of all other Ig isotypes [27]. Autoantibodies against most endogenous proteins have been found in mammalian blood and, interestingly, autoantibodies against Aβ, cellular enzymes, glial markers, lipid molecules, neurotransmitters and related receptors, tau, and vasculature-related molecules have been found altered in AD patients [28]. Given the proposed link between IAPP and AD pathology, we hypothesize that AD patients with IAPP brain pathology, just like T2D patients, demonstrate altered levels of IAPP-autoantibodies. Since, in a previous study, we have shown an *APOE* allele-dependent association between plasma IgA levels and AD pathology [29], and regarding the proposed pivotal role for APOE in brain IAPP accumulation, aggregation, and vasculopathy, we further found it interesting to investigate if IAPP-autoantibody levels are affected by *APOE4* status.

## 2. Results

### 2.1. Plasma IAPP-Autoantibody Levels in Relation to AD Pathology 

**Figure 1:**
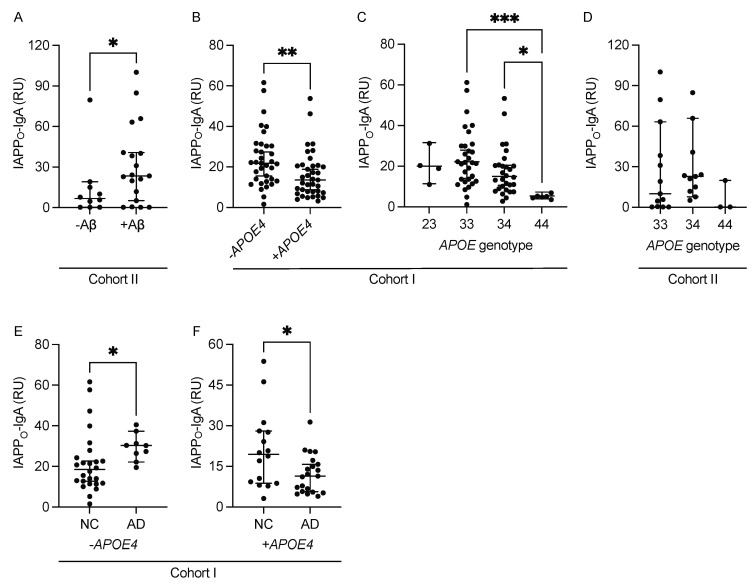
Plasma levels of immunoglobulin A (IgA) against islet amyloid polypeptide (IAPP) oligomers. The graphs illustrate significantly higher IAPP_O_-IgA levels in Aβ-positive (+Aβ) cases compared with Aβ-negative (−Aβ) in Cohort II (**A**); significantly lower IAPP_O_-IgA levels in Apolipoprotein E4 (*APOE4)* carriers (+*APOE4*) compared with non-carriers (−*APOE4*) in Cohort I (**B**); significantly lower IAPP_O_-IgA levels in *APOE44* carriers compared with *APOE33* and *APOE34* carriers in Cohort I (**C**); unaltered (but trending towards lower) IAPP_O_-IgA levels in *APOE44* carriers compared with *APOE33* and *APOE34* carriers in Cohort II (**D**); significantly higher IAPP_O_-IgA levels in Alzheimer’s disease (AD) patients compared with non-demented controls (NC) in *APOE4* non-carriers in Cohort I (**E**); and significantly lower IAPP_O_-IgA levels in AD patients compared with NC in *APOE4* carriers in Cohort I (**F**). Data were analyzed with either Mann–Whitney or Kruskal–Wallis tests and are presented as median with a 95% confidence interval. * Significant at *p* ≤ 0.05 level. ** Significant at *p* ≤ 0.01 level. *** Significant at *p* ≤ 0.001 level.

The study was initiated by measuring levels of plasma IgA, IgG, and IgM against IAPP monomers (IAPP_M_-Ig) and IAPP oligomers (IAPP_O_-Ig). We did not find any differences in levels of any of the IAPP-Igs between non-demented controls (NC) and AD patients in Cohort I (Table S1). In Cohort II, only IAPP_O_-IgA levels were significantly higher in +Aβ cases compared with −Aβ (Figure 1A, Table S2). However, this significance was lost after controlling for T2D, and the levels of the rest of the IAPP-Igs remained indifferent between +Aβ and −Aβ cases after the correction (Table S2). Levels of none of the IAPP-Igs differed between males and females in Cohort I (Table S3) and Cohort II (Table S4) regardless of controlling for T2D.

### 2.2. Plasma IAPP-Autoantibody Levels in Relation to APOE4 Status

Next, we investigated the difference in plasma IAPP-autoantibody levels in *APOE4* carriers and non-carriers. In Cohort I, the levels of IAPP_O_-IgA were significantly lower in *APOE4* carriers compared with non-carriers (Figure 1B, Table S1). In addition, there was an *APOE4* allele-dependent effect, where *APOE44* carriers had significantly lower IAPP_O_-IgA levels compared with *APOE33* and *APOE34* carriers (Figure 1C). None of the other IAPP-Igs (i.e., IAPP_M_-IgA, IAPP_M_-IgG, IAPP_O_-IgG, IAPP_M_-IgM, and IAPP_O_-IgM) differed between *APOE4* carriers and non-carriers (Table S1) or demonstrated an allele-dependent effect (Table S5). In Cohort II, none of the Igs differed between *APOE4* carriers and non-carriers (Table S2), and although a similar trend toward an *APOE4* allele-dependent effect was noted regarding IAPP_O_-IgA levels (Figure 1D), this trend was not significant either before or after controlling for T2D (*p* = 0.145 vs. *p* = 0.515, respectively). 

To further investigate the impact of *APOE4* status, we stratified Cohort I into *APOE4* carriers and non-carriers. Both IAPP_O_-IgA (Figure 1E) and IAPP_O_-IgM (227.38 ± 83.37 vs. 205.37 ± 241.17, *p* = 0.023, respectively) levels were significantly higher in AD patients compared with NC in *APOE4* non-carriers. In contrast, in *APOE4* carriers, the IAPP_O_-IgA levels were significantly lower in AD patients compared with NC (Figure 1F). In addition, the levels of IAPP_O_-IgA and IAPP_M_-IgA in *APOE4*-carrying AD patients were significantly lower compared with AD patients not carrying the *APOE4* allele (12.16 ± 7.14 vs. 29.46 ± 6.67, *p* < 0.001 and 5.95 ± 3.48 vs. 10.84 ± 5.72, *p* = 0.009, respectively). The levels of IAPP-IgM and IAPP-IgG were unaffected when comparing *APOE4* carriers and non-carriers regardless of AD diagnosis or IAPP aggregation status (Table S6). 

### 2.3. Plasma IAPP Levels in Cohort I and II

To further investigate if *APOE4* status influences plasma IAPP levels, we further analyzed the previously measured IAPP levels in Cohort I [23] and measured IAPP levels in post mortem-collected plasma of Cohort II. As previously described, plasma IAPP levels in Cohort I did not differ significantly between NC and AD patients (Table S7) [23]. In addition, we found no significant differences in plasma IAPP levels between *APOE4* carriers and non-carriers (321.33 ± 181.15 vs. 245.01 ± 135.37, respectively, *p* = 0.138). Interestingly, IAPP levels were close to significantly higher in AD patients compared with NC in *APOE4* non-carriers (319.37 ± 182.17 vs. 219.27 ± 107.80, respectively, *p* = 0.061), while IAPP levels in *APOE4*-carrying AD patients and NC were unchanged (315.72 ± 153.68 vs. 328.70 ± 217.18, respectively, *p* = 0.751). The plasma IAPP levels in Cohort II did not differ significantly between +Aβ and −Aβ cases (Table S8) or between *APOE4* carriers and non-carriers (253.37 ± 34.81 vs. 244.29 ± 18.66, respectively, *p* = 0.914), regardless of controlling for T2D (*p* = 0.390 and *p* = 0.436, respectively).

### 2.4. Brain IAPP Levels in Cohort II 

To investigate the relationship between *APOE4* status and IAPP in the brain, we next analyzed IAPP levels in the soluble fraction (IAPP-SF) and the insoluble fraction (IAPP-IF) of brain homogenates from cases in Cohort II. Neither the brain IAPP-SF levels nor IAPP-IF levels differed between +Aβ and −Aβ cases or between *APOE4* carriers and non-carriers (Table S2). After controlling for T2D, the brain IAPP-SF and IAPP-IF levels still did not differ between +Aβ and −Aβ cases or between *APOE4* carriers and non-carriers (Table S2). The visual representation of brain IAPP-SF and IAPP-IF of −Aβ and +Aβ cases can be found in Figure S1.

### 2.5. Correlations with Plasma IAPP-Autoantibody Levels

**Figure 2:**
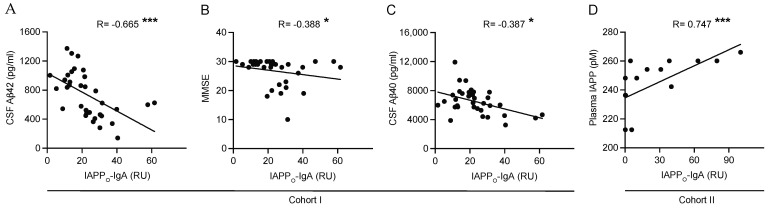
Correlations between plasma IAPP_O_-IgA levels and cognition, plasma IAPP levels, and levels of CSF AD biomarkers Aβ40 and Aβ42 in *APOE4* non-carriers. The graphs illustrate a significant negative correlation between plasma IAPP_O_-IgA levels and CSF Aβ42 levels in Cohort I (**A**); a significant negative correlation between plasma IAPP_O_-IgA levels and MMSE scores in Cohort I (**B**); a significant negative correlation between plasma IAPP_O_-IgA levels and CSF Aβ40 levels in Cohort I (**C**); and a significant positive correlation between plasma IAPP_O_-IgA levels and plasma IAPP levels in Cohort II (**D**); all in *APOE4* non-carriers. Data were analyzed using Spearman’s correlation test. * Significant at *p* ≤ 0.05 level. *** Significant at *p* ≤ 0.001 level.

Next, we analyzed the correlations between the plasma IAPP-autoantibody levels and memory test scores, CSF AD biomarker levels, CRP levels, plasma IgA levels, plasma IAPP levels, brain IAPP levels, and neuropathological scoring. The levels of IAPP-IgA (both IAPP_M_-IgA and IAPP_O_-IgA) correlated with total IgA in all groups, *APOE4* non-carriers, and *APOE4* carriers in both cohorts (Table 1). In *APOE4* non-carriers of Cohort I, both IAPP_M_-IgA and IAPP_O_-IgA correlated with CRP, CSF Aβ42, and a CSF Aβ42/40 ratio (Table 1, Figure 2A). The IAPP_O_-IgA also correlated with MMSE and CSF Aβ40 in these individuals (Table 1, Figure 2B,C). In *APOE4* carriers, only CSF Aβ42/40 correlated with IAPP_O_-IgA (Table 1). In Cohort II, both IAPP_M_-IgA and IAPP_O_-IgA correlated with plasma IAPP in all groups and in *APOE4* non-carriers (Table 1, Figure 2D). In addition, brain NFT scores correlated with IAPP_O_-IgA in all groups and with IAPP_M_-IgA in both *APOE4* non-carriers and carriers (Table 1). Lastly, brain IAPP-SF correlated with IAPP_O_-IgA in all groups and with both IAPP_O_-IgA and IAPP_M_-IgA in *APOE4* non-carriers (Table 1).

In Cohort I, levels of both IAPP_M_-IgG and IAPP_O_-IgG correlated with AQT and plasma IAPP levels. When stratified upon *APOE4* status, *APOE4* non-carriers demonstrated correlations between both IAPP_M_-IgG and IAPP_O_-IgG and plasma IAPP as well as CSF Aβ42/40 (Table 2). IAPP_M_-IgG also correlated with CSF Aβ42 (Table 2). In *APOE4* carriers, both IAPP_M_-IgG and IAPP_O_-IgG correlated with AQT, and IAPP_O_-IgG correlated with plasma IAPP (Table 2). Finally, in Cohort II, IAPP_M_-IgG levels correlated with plasma IAPP levels in all groups and in *APOE4* non-carriers, and with brain Aβ scores exclusively in *APOE4* carriers (Table 2). In addition, IAPP_M_-IgG levels correlated with brain IAPP-SF in *APOE4* non-carriers, and IAPP_O_-IgG correlated with brain IAPP-IF in *APOE4* carriers (Table 2).

Finally, in Cohort I, levels of IAPP_O_-IgM correlated with CSF Aβ42 and CSF Aβ42/40 in all groups (Table 3). In *APOE4* non-carriers, levels of both IAPP_M_-IgM and IAPP_O_-IgM correlated with CSF p-tau (Table 3). The IAPP_O_-IgM also correlated with MMSE, ADAS-Cog, CSF Aβ42, and CSF Aβ42/40 (Table 3). These correlations were not found in *APOE4* carriers (Table 3). Levels of IAPP_M_-IgM in all groups of Cohort II were associated with increased plasma IAPP but lowered brain IAPP-SF and IAPP-IF. The two former correlations were also found in *APOE4* non-carriers, but the correlation with brain IAPP-IF was no longer significant in the *APOE4* group (Table 3).

## 3. Discussion

The current study aimed to investigate the presence of autoantibodies against monomeric and oligomeric IAPP to further narrow down whether alterations in peripheral production, aggregation, or/and clearance of IAPP are implicated in AD. Our analysis showed that neither IAPP-IgG nor IAPP-IgM levels differed between AD and NC in Cohort I or between +Aβ and −Aβ cases in Cohort II. In contrast, an increase in IAPP_O_-IgA levels was detected in +Aβ cases compared with −Aβ in Cohort II, but this increase was not found in the larger Cohort I. Hence, at a first glance, it appears as if an alteration in monomeric or oligomeric IAPP levels is not implicated in AD and neither is the availability of Igs against the different IAPP aggregation forms. However, when we divided the cohorts based on *APOE4* status (a well-known AD risk factor), the *APOE4* carriers in Cohort I demonstrated significantly lower IAPP_O_-IgA levels compared with non-carriers. Interestingly, this reduction was not seen in IAPP_M_-IgA levels or levels of the other two Ig isotypes (regardless of IAPP aggregation status), suggesting that the IgA clearance of IAPP_O_ is specifically affected in *APOE4* carriers. The largest reduction was seen in homozygous *APOE4* carriers, emphasizing the impact of the *APOE* polymorphism on the IAPP_O_-IgA levels. 

The underlying cause to this *APOE4*-dependent reduction in IAPP_O_-IgA levels is difficult to speculate upon, as the literature lacks studies investigating the relationship between *APOE* isoforms and IAPP-autoantibodies. However, there are a few studies on Ig in knock-in mice expressing human *APOE* that may be instructive to consider. For instance, a smaller number of antibody-producing B cells has been found in the spleen and blood of *APOE4*-transgenic mice compared with mice expressing *APOE3* [30], which could, in turn, result in lower levels of antibodies in general. Another study has demonstrated lower total IgG and IgA levels in the spleen of *APOE4* knock-in mice compared with *APOE3*, but the levels of IgG2a subtype and IgM were quite high in *APOE4* mice, suggesting differential Ig class switching in *APOE4* mice compared with *APOE3* or *APOE2* mice [31]. Since cytokines secreted by T helper cells can alter B cell isotype switching, the modulation of cytokine profile by *APOE* genotype [32,33] may be responsible for the observed alteration in Ig expression. Interestingly, in the same study, the blood Ig levels seemed to be unaltered in the *APOE4* mice compared with *APOE3* (except from IgG2a, which was significantly higher in *APOE4* mice compared with *APOE2* and *APOE3* mice) [31]. Thus, it appears as if the IgA production in peripheral organs (e.g., bone marrow, spleen, lymph nodes) is, to some degree, *APOE* allele-dependent, but this relation cannot be detected in blood. We have recently published a study where we demonstrated unaltered levels of plasma total IgA between *APOE4* carriers and non-carriers [29] in the individuals included in Cohort I of the current study. This finding supports the previous results of the *APOE* mice study, i.e., that *APOE4* status does not affect IgA levels in the blood. Hence, we draw the conclusion that the reduced signal yielded in our IAPP_O_-IgA ELISA is not due to a reduction in the total IgA production. Therefore, we next investigated if the phenomenon was due to alterations in plasma IAPP levels. Although we were unable to detect significant differences in plasma IAPP levels between *APOE4* carriers and non-carriers, we did note a trend toward increased IAPP levels in *APOE4* carriers in Cohort I. Furthermore, the association analysis showed that plasma IAPP levels correlated positively with IAPP-IgG in Cohort I and IAPP-IgA, IAPP_M_-IgG, and IAPP_M_-IgM in Cohort II. We thus conclude that the reduction in IAPP_O_-IgA levels in *APOE4* carriers is not due to a reduced amount of circulating IAPP. Instead, we speculate that the slightly higher plasma IAPP levels in *APOE4* carriers and AD patients are due to a reduced removal of IAPP. An alternative scenario is that it is not the production of IgA or IAPP that is altered, but rather the affinity of IgA to bind IAPP. Normally, in biological fluids, both antigens and antibodies are in dynamic equilibrium between unbound and bound forms in a concentration-dependent manner. Therefore, the antigen may mask a proportion of the corresponding antibody and limit the detection of both. Such an increase in IAPP-antibody binding (or a decrease in IAPP-antibody detection) could be due to the slightly larger amounts of IAPP found in *APOE4* carriers, but also potentially due to larger proportions of oligomeric IAPP in these individuals, as antibodies in general bind oligomers with a much higher affinity compared with monomers [34]. 

Another alternative explanation for the lowered IAPP_O_-IgA levels in *APOE4* carriers is linked to epitope exposure. The role of APOE4 in amyloid plaque formation in the brain parenchyma and vessel walls (cerebral amyloid angiopathy) has been repeatedly studied [35,36], and several studies suggest that the binding of APOE to Aβ is implicated in Aβ aggregation [36]. The binding appears to be Aβ aggregation status- and APOE isotype-dependent, as experimental studies show that APOE binds Aβ oligomers rather than monomers and that the interaction with APOE4 is stronger compared with that of APOE3 [37]. Interestingly, AD patients carrying at least one *APOE4* allele demonstrate lower levels of Aβ42-autoantibodies [38], which in theory could be due to a masked epitope caused by APOE4–Aβ oligomer binding. Since APOE also binds to oligomeric IAPP in preference to the monomeric IAPP [22], we speculate that APOE4 in the plasma binds to the IAPPo in the ELISA, and thereby mask the IgA specific IAPP_O_ epitopes. Finally, IAPP_M_ is a very small peptide (37 amino acids) with few epitopes (presumably 1–3 epitopes). When IAPP_M_ oligomerizes, in analogy to all aggregation, some of its epitopes get hidden. If the epitopes of the peptide are located within such an area, then the antibodies directed against it can no longer bind. Hence, the absolute signal in our ELISA could be dependent on the IAPP epitope availability, provided that IAPP epitopes in *APOE4* carriers differ from IAPP epitopes in *APOE2* and *APOE3* carriers.

**Figure 3:**
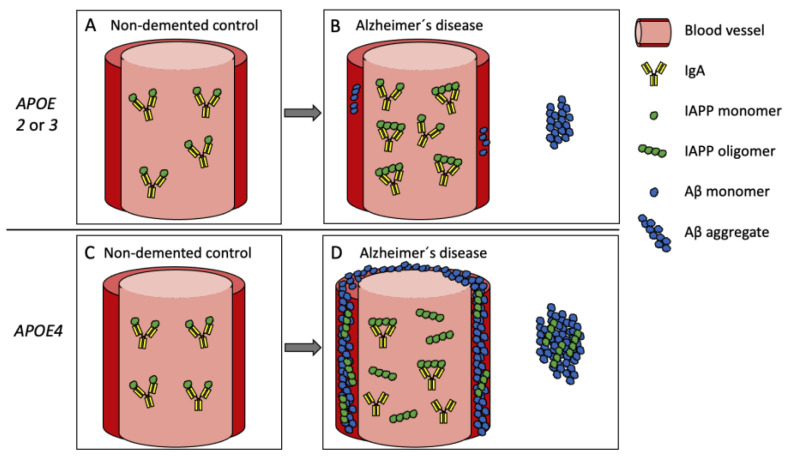
Simplified illustration of a hypothesis describing the relationship between IgA, *APOE*, IAPP, and Aβ. Plasma IAPP in healthy individuals (**A**,**C**) is sufficiently removed by circulating autoantibodies, including IgA. The amount of circulating IAPP (both monomers and oligomers) increases in Alzheimer’s disease (**B**,**D**), but they are removed by the compensating increase in levels of IgA directed against IAPP oligomers (**B**). The compensatory effect is lost in *APOE4* carriers (**D**) either due to epitope masking by APOE or aggregation or from increased levels of oligomeric IAPP, which leaves the brain exposed to a higher influx of IAPP oligomers. The incoming IAPP oligomers seed amyloid beta (Aβ) and accelerate amyloid deposition in vessel walls and brain parenchyma.

The levels of IAPP_O_-IgA (and IAPP_O_-IgM) were significantly higher in *APOE4*-non-carrying AD patients compared with controls whereas, in *APOE4*-carrying AD patients, the IAPP_O_-IgA levels were decreased. In view of our discussion above, we interpret this finding as evidence for increased levels of circulating IAPP_M_, and thereby the levels of autoantibodies against the peptide, in *APOE4*-non-carrying AD patients. This AD-related increase is masked in *APOE4*-carrying AD patients either due to higher plasma IAPPo levels, increased APOE4–IAPP binding, or reduced production of specifically IAPP_O_-IgA due to epitope masking. All scenarios would implicate a reduction in IAPP_O_ clearance in *APOE4*-carrying AD patients. These scenarios should also be considered when interpreting the results we obtained after analyzing correlations between IAPP-Ig levels (in particular IAPP-IgA) and AD pathology-associated variables. The correlations were, to a large extent, only found in *APOE4* non-carriers, which may again be explained by masked epitopes or increased plasma IAPP_O_ levels in *APOE4* carriers. Nevertheless, the significant correlations found between IAPP-Igs and AD markers, as well as cognitive test results, highlight the implication of IAPP in AD. In particular, the negative correlation between IAPP-Igs and CSF Aβ (Aβ40 or Aβ42) in *APOE4* non-carriers is of interest as it supports the idea that IAPP and Aβ pathology is interlinked. This is exemplified in studies demonstrating co-depositions of IAPP and Aβ in the brain [14] and IAPP seeding Aβ under experimental conditions [39]. Finally, the negative correlations between nearly all IAPP-Igs and brain IAPP-SF levels support the idea that altered IAPP binding and clearance by IAPP-Igs lead to an increased exposure and influx of IAPP into the brain. If this holds true, a reduction of IAPP_O_ clearance by IgA in *APOE4* homozygotes may have detrimental consequences, where the increased amount of incoming IAPP_O_ accelerates Aβ seeding and deposition of Aβ and IAPP in vessel walls (a simplified illustration of the theory is found in Figure 3).

Our analysis of brain IAPP-SF and IAPP-IF, however, did not show significant differences between *APOE4* carriers and non-carriers; hence, we were unable to find support for this idea with the methods we used. Notably, the analysis of IAPP levels in the brain was performed on paraformaldehyde (PFA)-fixed brain tissue. This is a limitation of the study as PFA-fixed tissue is more difficult to homogenize, and the fixation itself may compromise antigen exposure. Hence, studies on fresh-frozen tissue are warranted to further explore the theory and investigate the link between plasma IgA and IAPP depositions in the brain. There are also other methodological limitations which need to be addressed. Firstly, in experimental conditions, IAPP forms aggregates very rapidly. Hence, although we carefully characterized our IAPP preparations, we cannot exclude the possibility that the IAPP_M_ and IAPP_O_ preparations used in our indirect ELISA also contained unwanted aggregation variants, including monomers, oligomers, and fibrils. Secondly, the fact that the plasma samples were collected in clinical routine without fasting prescriptions most likely influences our results. Thus, to fully evaluate potential correlations between IAPP-autoantibodies and IAPP levels, studies on plasma samples collected with fasting prescriptions are warranted. It should further be emphasized that Cohort II is a rather small cohort consisting of several different cases with dementia diagnosis and T2D of which only n = 3 were *APOE4* homozygotes. Studies on larger cohorts are highly warranted to further understand the capacity of IAPP-Igs to clear circulatory IAPP and accumulation of IAPP in the brain. Finally, the plasma from Cohort II was collected post mortem, and we cannot exclude the possibility that processes occurring after death may have affected the plasma we have analyzed. These limitations, which are mostly related to Cohort II, may have contributed to discrepancies between the analyzed cohorts. 

## 4. Materials and Methods

### 4.1. Individuals Included in the Study

In this study, we analyzed plasma samples collected ante mortem (Cohort I) and post mortem (Cohort II). Cohort I, consisting of AD patients (n = 30) and healthy age-matched controls (NC, n = 42), was described in a previous study; thus, the demographic data, performance during cognitive tests, Q-albumin, and levels of plasma C-reactive protein (CRP), CSF Aβ40, CSF Aβ42, CSF p-tau, CSF t-tau, plasma IgA, and plasma IAPP have been published previously [23,29]. Both controls and AD patients underwent cognitive and neurological assessments at the Memory Clinic at Skåne University Hospital, Sweden, by a physician with a special interest in dementia disorders. Patients with AD were diagnosed according to the DSM-IV criteria for Alzheimer’s disease. The cognitively healthy individuals displayed no neurological or cognitive deficiency symptoms. The demographic data and mean values of the variables can be found in Table S7. Cohort II (n = 29) consisted of histopathologically evaluated donors from The Netherlands Brain Bank (NBB). The cohort included AD patients (n = 16), NC (n = 7), multiple sclerosis (MS) patients (n = 3), a vascular dementia (VaD) patient (n = 1), a frontotemporal dementia (FTD) patient (n = 1), and a patient with hippocampal alterations (n = 1). Of these cases, n = 8 individuals were diagnosed with T2D. The presence of Aβ plaques was scored into O, A, B, and C, where O = zero, A = some, B = moderate, and C = many, and the presence of NFT and neuropil threads was scored into I–VI according to Braak [40]. The demographic data and mean values of the variables are described in Table S8. The demographic data, T2D status, neuropathological evaluation, and cause of death can be found in Table S9. Informed consent for the use of brain tissue, plasma, and clinical data for research purposes was obtained from all subjects or their legal representatives in accordance with the International Declaration of Helsinki. 

### 4.2. Stratification of Cohorts

Cohort I was stratified upon NC and AD groups, while Cohort II was stratified upon Aβ-negative (−Aβ, n = 10) and Aβ-positive (+Aβ, n = 19) cases. The −Aβ group consisted of cases with Braak Aβ stages O to A, while the +Aβ group consisted of Braak Aβ stages B to C. All cases in the −Aβ group demonstrated Braak NFT stages 0 to 3. Both cohorts were also stratified into *APOE4* non-carriers and *APOE4* carriers. Individuals with genotypes *APOE23* (n = 4 in Cohort I and n = 2 in Cohort II) and *APOE33* (n = 31 in Cohort I and n = 13 in Cohort II) were stratified as *APOE4* non-carriers. Individuals with *APOE24* (n = 2 in Cohort I) as well as *APOE34* (n = 29 in Cohort I and n = 11 in Cohort II) and *APOE44* (n = 6 in Cohort I and n = 3 in Cohort II) were stratified as *APOE4* carriers. In Cohort I, we further divided *APOE4* non-carriers or carriers upon NC and AD; however, in Cohort II, we did not stratify *APOE4* non-carriers or carriers upon −Aβ and +Aβ due to the small sample size. 

### 4.3. IAPP Preparation

The IAPP monomers were prepared by dissolving the lyophilized human IAPP_1-37_ peptide (AlexoTech, Umeå, Sweden) in dimethyl sulfoxide to a concentration of 2.5 mM, water-sonicating for 10 min, and further diluting with Dulbecco’s phosphate-buffered saline (DPBS) to a concentration of 100 μM. The IAPP oligomers were prepared by solubilizing the lyophilized human IAPP_1-37_ peptide in 20 mM sodium hydroxide (pH 12). The pH was adjusted to pH 7 by diluting the solution in a phosphate buffer to a concentration of 100 μM. Thereafter, the IAPP preparation was agitated for 20 min at room temperature (RT), followed by centrifugation at 14,000× *g* for 10 min (Biofuge 13, Heraeus Sepatech) at 4 °C. The lower fraction (50 μL) was collected and stored at −20 °C. Before use, the concentration of IAPP oligomers was evaluated with a Pierce BCA Protein Assay Kit (Thermo Scientific, Rockford, IL, USA). Both the monomeric and oligomeric IAPP preparations were evaluated by Western blot using rabbit anti-IAPP *A133 antibody* (a kind gift from Gunilla Westermark, Uppsala University, Sweden) to confirm the presence of the respective aggregation variants (Figure S2). 

### 4.4. Analysis of Plasma IAPP

The plasma IAPP levels were measured using a Human Amylin ELISA kit (EZHA-52K, Merck, Sweden) according to manufacturer’s instructions.

### 4.5. Analysis of Plasma IAPP-Autoantibodies

Autoantibodies were detected by an in-house developed indirect ELISA based on published protocols used in previous IAPP-autoantibody studies [24,25]. Optically clear 96-well flat bottom microplates (Nunc, Thermo Scientific, Roskilde, Denmark) were coated with either IAPP monomers or oligomers at a concentration of 1 mg/L in PBS and incubated overnight at 4 °C. The plates were then washed three times with 0.05% Tween in PBS (PBS-T). Non-specific binding sites on the plastic were blocked with 1% bovine serum albumin (BSA, Merck, Darmstadt, Germany) in 0.025% PBS-T for 1h at RT and thereafter washed three times with PBS-T. Plasma samples were diluted 1:60–640 with 1% BSA in PBS-T and incubated for 2 h at RT with agitation. Following incubation, plates were washed five times with PBS-T. Antibody binding was detected with horseradish peroxidase (HRP)-conjugated polyclonal rabbit anti-human IgA, IgG, or IgM (DakoCytomation, Glostrup, Denmark) diluted with 1% BSA in PBS-T and incubated for 1h at RT with agitation. After three washes with PBS-T, peroxidase substrate (SeraCare, Gaithersburg, MD, USA) was applied to each well, and the reaction was allowed to proceed in the dark for 10 min at RT. The reaction was terminated by the addition of 1M H_2_SO_4_. The end-point optical densities were read immediately at a wavelength of 450 nm on a microwell plate reader (BioTek, EON^TM^). All samples had respective BSA controls where the wells were coated with 1 mg/L BSA in PBS, and the procedure was followed as described above. Rabbit anti-human IAPP IgG (Peninsula Laboratories, San Carlos, CA, USA) diluted 1:500–1:32,000 and HRP-conjugated polyclonal goat anti-rabbit (DakoCytomation) were used to create a standard curve. Additionally, an inter-control was applied to estimate the reproducibility of the signal throughout the study (CV = 7.15). The IAPP-autoantibody levels were defined as relative units (RU).

### 4.6. Brain Homogenization and Protein Level Determination

Paraformaldehyde immersion-fixed brain tissue (10 mg) containing hippocampus and entorhinal cortex from cases included in Cohort II were homogenized at 10% (*w*/*v*) in 1% Triton X-100 in Tris-buffered saline (TBS) using Dounce homogenizers. The homogenates were thereafter water-sonicated for 10 min and centrifuged at 14,000× *g* for 30 min at 4 °C. The supernatant was collected and is hereon referred to as the soluble fraction (SF). The pellet was resuspended at 10% (*w*/*v*) in 70% formic acid in TBS, water-sonicated for 10 min, and centrifuged at 14,000× *g* for 1h at 4 °C. The supernatant, which is hereon referred to as the insoluble fraction (IF), was collected, neutralized at 1:20 with 1 M Tris-base (pH 9) at RT, and reduced using speed-vac. The protein concentration was estimated using a Pierce BCA Protein Assay Kit. 

### 4.7. Dot-Blot

Samples of brain IAPP-SF and IAPP-IF were normalized according to the BCA data, loaded (2 µL) onto a nitrocellulose membrane, and thereafter left to dry. The membrane was then washed with 0.05% PBS-T for 10 min, blocked with 1% BSA in PBS-T for 1h at RT, and incubated with rabbit-anti-human A133 antibody in blocking solution overnight at 4 °C with agitation. The membrane was then washed in PBS-T for 10 min three times and incubated with HRP-conjugated goat-anti-rabbit antibody (DakoCytomation, Glostrup, Denmark) in blocking solution for 1h at RT with agitation. The membrane was washed in PBS-T for 10 min three times, in PBS for 10 min once, and visualized using Luminata Forte Western HRP Substrate (Millipore, Darmstadt, Germany) and ChemiDoc XRS1 System (BioRad, Hercules, CA, USA) (Figure S1). The intensity of dots in digitalized images of immunoblotted membranes was analyzed using ImageJ 1.53a (National Institutes of Health, USA). The brain IAPP-SF and IAPP-IF levels were defined as relative units (RU). 

### 4.8. Statistical Analyses

All statistical analyses were performed using the SPSS software (version 28). The Kolmogorov–Smirnov test was used to assess normal distribution. The normally distributed data (age, CSF Aβ40, total IgA in Cohort I and PMD, IAPP_M_-IgA, and total IgA in Cohort II) were analyzed using Student’s t-test or one-way ANOVA. The skewed data (MMSE, ADAS-Cog, AQT, plasma IAPP, CRP, Q-albumin ratio, CSF Aβ42, CSF Aβ42/40 ratio, CSF p-tau, CSF t-tau, IAPP_M_-IgA, IAPP_O_-IgA, IAPP_M_-IgM, IAPP_O_-IgM, IAPP_M_-IgG, IAPP_O_-IgG in Cohort I and age, brain Aβ score, brain NFT score, IAPP_M_-IgM, IAPP_M_-IgG, IAPP_O_-IgA, IAPP_O_-IgM, IAPP_O_-IgG, plasma IAPP, brain IAPP-SF levels, brain IAPP-IF levels in Cohort II) were analyzed using Mann–Whitney or Kruskal–Wallis tests. The Cohort II data were further analyzed using a Univariate Linear Model where T2D was included as a covariate. The association analyses between the investigated variables were performed using the 2-tailed Spearman’s correlation test. The correlations and differences were considered significant at *p* ≤ 0.05.

## 5. Conclusions

Our study demonstrates an *APOE4* allele-dependent decrease in IAPP_O_-IgA levels and that IAPP-Igs are associated with AD pathology biomarkers and cognitive decline, specifically in *APOE4* non-carriers. These findings suggest that IAPP_O_ and autoantibodies against the peptide are implicated in AD-related events in an *APOE4-*dependent manner, potentially driven by an enhanced influx of toxic IAPP_O_ from blood to brain due to a reduced clearance by IAPP_O_-Igs. Further studies investigating the role of *APOE4* and Igs (in particular IgA) in IAPP_O_ clearance are warranted.

## Figures and Tables

**Table 1:** Correlations between plasma IAPP-IgA levels and variables associated with AD pathology.

	IAPP_M_-IgA (RU)			IAPP_O_-IgA (RU)		
	All Groups	−*APOE4*	+*APOE4*	All Groups	−*APOE4*	+*APOE4*
Cohort I:						
Total IgA (mg/mL)	0.658 ***	0.813 ***	0.450 **	0.589 ***	0.845 ***	0.350 *
MMSE (score)	ns	ns	ns	ns	−0.388 *	ns
CRP (mg/mL)	ns	0.532 **	ns	ns	0.561 ***	ns
CSF Aβ40 (pg/mL)	ns	ns	ns	ns	−0.387 *	ns
CSF Aβ42 (pg/mL)	ns	−0.510 **	ns	ns	−0.665 ***	ns
CSF Aβ42/40	ns	−0.479 **	ns	ns	−0.575 ***	0.378 *
Cohort II:						
Total IgA (mg/mL)	0.750 ***	0.846 ***	0.556 *	0.802 ***	0.850 ***	0.634 *
Brain NFT (score)	ns	0.555 *	−0.550 *	0.387 *	ns	ns
Plasma IAPP (pM)	0.576 ***	0.769 ***	ns	0.521 **	0.747 ***	ns
Brain IAPP-SF (RU)	ns	−0.692 **	ns	−0.404 *	−0.629 *	ns

Data were analyzed using Spearman’s correlation test. Aβ—amyloid beta, *APOE*—apolipoprotein E, CRP—C-reactive protein, CSF—cerebrospinal fluid, IAPP—islet amyloid polypeptide, Ig—immunoglobulin, M—monomer, MMSE—Mini-Mental State Examination, NFT—neurofibrillary tangle, ns—not significant, O—oligomer, RU—relative unit, SF—soluble fraction. * Significant at *p* ≤ 0.05 level. ** Significant at *p* ≤ 0.01 level. *** Significant at *p* ≤ 0.001 level.

**Table 2:** Correlations between plasma IAPP-IgG levels and variables associated with AD pathology.

	IAPP_M_-IgG			IAPP_O_-IgG		
	All Groups	−*APOE4*	+*APOE4*	All Groups	−*APOE4*	+*APOE4*
Cohort I:						
AQT (score)	0.261 *	ns	0.382 *	0.267 *	ns	0.428 *
Plasma IAPP (pM)	0.288 *	0.356 *	ns	0.436 ***	0.501 **	0.431 **
CSF Aβ42 (pg/mL)	ns	−0.395 *	ns	ns	ns	ns
CSF Aβ42/40	ns	−0.415 *	ns	ns	−0.419 *	ns
Cohort II:						
Plasma IAPP (pM)	0.617 ***	0.729 **	ns	ns	ns	ns
Brain Aβ (score)	ns	ns	−0.541 *	ns	ns	ns
Brain IAPP-SF (RU)	ns	−0.546 *	ns	ns	ns	ns
Brain IAPP-IF (RU)	ns	ns	ns	ns	ns	−0.556 *

Data were analyzed using Spearman’s correlation test. Aβ—amyloid beta, *APOE*—apolipoprotein E, AQT—A Quick Test, CSF—cerebrospinal fluid, IAPP—islet amyloid polypeptide, IF—insoluble fraction, Ig—immunoglobulin, M—monomer, ns—not significant, O—oligomer, RU—relative unit, SF—soluble fraction. * Significant at *p* ≤ 0.05 level. ** Significant at *p* ≤ 0.01 level. *** Significant at *p* ≤ 0.001 level.

**Table 3:** Correlations between plasma IAPP-IgM levels and variables associated with AD pathology.

	IAPP_M_-IgM			IAPP_O_-IgM		
	All Groups	−*APOE4*	+*APOE4*	All Groups	−*APOE4*	+*APOE4*
Cohort I:						
MMSE (score)	ns	ns	ns	ns	−0.459 **	ns
ADAS-Cog (score)	ns	ns	ns	ns	0.480 **	ns
CSF p-tau (pg/mL)	ns	0.363 *	ns	ns	0.448 **	ns
CSF Aβ42 (pg/mL)	ns	ns	ns	−0.242 *	−0.430 *	ns
CSF Aβ42/40	ns	ns	ns	−0.285 *	−0.524 ***	ns
Cohort II:						
Plasma IAPP (pM)	0.511 **	0.843 ***	ns	ns	ns	ns
Brain IAPP-SF (RU)	−0.420 *	−0.543 *	ns	ns	ns	ns
Brain IAPP-IF (RU)	−0.424 *	ns	ns	ns	ns	ns

Data were analyzed using Spearman’s correlation test. Aβ—amyloid beta, ADAS-Cog—Alzheimer’s Disease Assessment Scale—Cognitive Subscale, *APOE*—apolipoprotein E, CSF—cerebrospinal fluid, IAPP—islet amyloid polypeptide, IF—insoluble fraction, Ig—immunoglobulin, M—monomer, MMSE—Mini-Mental State Examination, ns—not significant, O—oligomer, p-tau—phosphorylated tau, RU—relative unit, SF—soluble fraction. * Significant at *p* ≤ 0.05 level. ** Significant at *p* ≤ 0.01 level. *** Significant at *p* ≤ 0.001 level.

## Data Availability

The data presented in this study are available on request from the corresponding author. The data are not publicly available due to ethical restrictions.

## Supplementary Materials

The following supporting information can be downloaded at: https://www.mdpi.com/article/10.3390/ijms24043776/s1.
