# Supplementary material for: Plasma IAPP-Autoantibody Levels in Alzheimer’s Disease Patients Are Affected by APOE4 Status
Source: Int J Mol Sci. 2023 Feb 14;24(4):3776. doi: 10.3390/ijms24043776 (PMC9960837; doi:10.3390/ijms24043776)
Supplement: Supplementary file 1 [file ijms-24-03776-s001.zip › ijms-2195058-supplementary.pdf]

Table S1. Plasma IAPP-Ig levels in Cohort I.

IAPP-Ig (RU):	NC (n=42)	AD (n=30)	-*APOE4* (n=35)	+*APOE4* (n=37)
IAPP$_M$-IgG	46.5±29.9	48.1±24.2	51.0±30.1	43.5±24.6
IAPP$_O$-IgG	70.6±58.0	64.6±26.3	75.0±63.0	61.6±23.9
IAPP$_M$-IgM[a]	158.2±83.1	177.8±96.4	162.9±74.9	170.1±101.3
IAPP$_O$-IgM	212.2±245.2	198.6±109.6	211.0±210.9	202.3±190.1
IAPP$_M$-IgA	8.1±5.2	7.4±4.8	8.5±5.5	7.2±4.4
IAPP$_O$-IgA	21.5±14.5	17.4±10.6	23.7±13.7	16.0±11.4**

[a]n=40 in NC. Data was analyzed using Mann-Whitney test and values are presented as mean value ± SD. AD – Alzheimer's disease, APOE – apolipoprotein E, IAPP – islet amyloid polypeptide, Ig – immunoglobulin, M – monomer, NC – non-demented control, O – oligomer, RU – relative unit. **Significant at p≤0.01 level.

Table S2. Plasma IAPP-Ig and brain IAPP levels in Cohort II.

	-Aβ (n=10)	+Aβ (n=19)	p-value*	-*APOE4* (n=15)	+*APOE4* (n=14)	p-value*
IAPP$_M$-IgG[a]	21.9±12.2	25.5±34.8	0.875	18.9±10.1	30.0±40.0	0.340
IAPP$_O$-IgG[a]	34.8±29.2	51.8±80.4	0.751	34.4±18.3	58.3±94.9	0.412
IAPP$_M$-IgM[a]	65.7±56.1	71.6±39.1	0.462	64.0±45.9	75.6±44.4	0.413
IAPP$_O$-IgM[a]	35.4±26.7	62.9±94.2	0.351	57.0±96.4	49.5±55.8	0.802
IAPP$_M$-IgA[a]	9.3±7.0	12.9±7.2	0.099	11.7±8.9	11.5±5.3	0.977
IAPP$_O$-IgA[a]	14.3±23.8	31.3±29.2*	0.174	26.3±32.3	24.5±24.4	0.802
IAPP-SF[b]	1.9±2.0	2.4±3.4	0.546	2.2±2.7	2.2±3.2	0.999
IAPP-IF[b]	0.4±0.0	0.4±0.1	0.441	0.4±0.1	0.4±0.1	0.948

[a]Plasma IAPP-Ig levels (RU). [b]Brain IAPP levels (RU). *Corrected for Type 2 diabetes. Data was analyzed using either Student's t-test or Mann-Whitney test, and values are presented as mean value ± SD. Aβ - amyloid beta, APOE – apolipoprotein E, IAPP – islet amyloid polypeptide, Ig – immunoglobulin, M – monomer, O – oligomer, RU – relative unit. *Significant at p≤0.05 level.

Table S3. Plasma IAPP-Ig levels in males and females in Cohort I.

IAPP-Ig (RU):	Males (n=22)	Females (n=50)
**IAPP$_M$-IgG**	49.4±26.1	46.2±28.3
**IAPP$_O$-IgG**	72.8±50.8	66.0±46.0
**IAPP$_M$-IgM[a]**	147.2±67.3	174.9±96.1
**IAPP$_O$-IgM**	212.0±217.4	204.2±192.8
**IAPP$_M$-IgA**	8.1±5.4	7.7±4.8
**IAPP$_O$-IgA**	21.6±15.6	19.0±11.9

[a]n=21 males and n=49 females. Data was analyzed using Mann-Whitney test and values are presented as mean ± SD. IAPP – islet amyloid polypeptide, Ig – immunoglobulin, M – monomer, O – oligomer, RU – relative unit.

Table S4. Plasma IAPP-Ig levels in males and females in Cohort II.

IAPP-Ig (RU):	Males (n=12)	Females (n=17)	p-value*
**$IAPP_M$-IgG**	18.3±6.1	28.5±37.1	0.356
**$IAPP_O$-IgG**	69.1±99.2	29.6±20.2	0.131
**$IAPP_M$-IgM**	73.4±49.5	66.9±42.4	0.675
**$IAPP_O$-IgM**	42.4±60.5	61.1±89.5	0.543
**$IAPP_M$-IgA**	10.8±5.8	12.2±8.2	0.631
**$IAPP_O$-IgA**	20.1±18.5	29.1±33.5	0.394

*Corrected for Type 2 diabetes. Data was analyzed using Mann-Whitney test and values are presented as mean ± SD. IAPP – islet amyloid polypeptide, Ig – immunoglobulin, M – monomer, O – oligomer, RU – relative unit.

Table S5. Plasma IAPP-Ig levels across *APOE* genotypes in Cohort I.

IAPP-Ig (RU):	*APOE23* (n=4)	*APOE33* (n=31)	*APOE24* (n=2)*	*APOE34* (n=29)	*APOE44* (n=6)
**$IAPP_M$-IgG**	41.0±7.5	52.3±31.8	19.7±21.2	44.5±23.2	46.5±31.7
**$IAPP_O$-IgG**	62.9±9.5	76.5±66.8	43.0±11.6	61.3±19.0	69.1±43.0
**$IAPP_M$-IgM[a]**	186.3±52.8	159.7±77.5	112.5±3.0	166.8±101.1	204.7±117.4
**$IAPP_O$-IgM**	144.3±29.9	219.6±222.9	91.5±30.6	204.2±206.1	230.5±127.2
**$IAPP_M$-IgA**	8.8±5.2	8.5±5.6	11.0±6.0	7.3±4.4	5.4±3.5

[a]n=30 *APOE33* and n=28 *APOE34*. *Not included in the Kruskal-Wallis test. Data was analyzed using Kruskal-Wallis test and values are presented as mean ± SD. APOE – apolipoprotein E, IAPP – islet amyloid polypeptide, Ig – immunoglobulin, M – monomer, O – oligomer, RU – relative unit.

Table S6. Plasma IAPP-Ig levels in non-demented controls and AD patients stratified upon the *APOE4* status in Cohort I.

	**NC**		**AD**	
**IAPP-Ig (RU):**	-*APOE4* (n=26)	+*APOE4* (n=16)	-*APOE4* (n=9)	+*APOE4* (n=21)
**$IAPP_M$-IgG**	48.1±29.5	43.9±31.2	59.6±32.1	43.1±18.8
**$IAPP_O$-IgG**	78.1±72.0	58.5±17.9	66.1±23.6	63.9±27.8
**$IAPP_M$-IgM[a]**	154.1±72.5	165.1±100.6	187.2±80.1	173.8±104.1
**$IAPP_O$-IgM**	205.4±241.2	223.4±259.1	227.4±83.4	186.3±118.7

[a]n=25 -*APOE4* and n=15 +*APOE4*. Data was analyzed using Mann-Whitney test and values are presented as mean ± SD. AD – Alzheimer's disease, APOE – apolipoprotein E, IAPP – islet amyloid polypeptide, Ig – immunoglobulin, M – monomer, NC – non-demented control, O – oligomer, RU – relative unit.

Table S7. Clinical data of Cohort I individuals stratified by AD diagnosis.

Variables:	NC, n=42	AD, n=30
Age (years)	74±6	74±7
APOE4 carriers, n (%)[a]	16/42 (38%)	21/30 (70%)
Females, n (%)[a]	29/42 (69%)	21/30 (70%)
MMSE (score)	29.19±0.83	19.50±4.26***
ADAS-Cog (score)	1.88±1.70	8.67±1.90***
AQT (sec)	63.24±10.36	110.18±46.72***
CRP (mg/l)	1.75±1.59	6.94±13.12*
Q-albumin	6.75±3.80	6.80±2.54
CSF Aβ40 (pg/ml)	6759.52±1906.93	6452.66±1675.20
CSF Aβ42 (pg/ml)	791.67±289.29	386.70±110.29***
CSF p-tau (pg/ml)	45.61±18.16	120.40±41.24***
CSF t-tau (pg/ml)	341.53±106.23	621.81±207.42***
Plasma IAPP (pM)	260.96±165.03	316.82±159.52
Total IgA (mg/ml)	3.44±1.58	4.21±1.52*

[a]Data is presented for illustrative purposes and was not analyzed. Data was analyzed using either Student's t-test or Mann-Whitney test, and values are presented as mean value ± SD. Aβ - amyloid beta, AD – Alzheimer's disease, ADAS-Cog – Alzheimer's Disease Assessment Scale – Cognitive Subscale, APOE – apolipoprotein E, AQT – A Quick Test, CRP – C-reactive protein, CSF – cerebrospinal fluid, IAPP – islet amyloid polypeptide, Ig – immunoglobulin, MMSE – Mini-Mental State Examination, NC – non-demented control, p-tau – phosphorylated tau, t-tau – total tau. *Significant at $p \leq 0.05$ level. ***Significant at $p \leq 0.001$ level.

Table S8. Clinical data of Cohort II cases stratified by brain Aβ pathology status.

Variables:	-Aβ, n=10	+Aβ, n=19
Age (years)	75±9	81±11
APOE4 carriers, n (%)[a]	2/10 (20%)	12/19 (63%)
T2D positive, n (%)[a]	5/10 (50%)	3/19 (16%)
Females, n (%)[a]	6/10 (60%)	11/19 (58%)
PMD (h)	7.33±2.01	6.18±1.39
Plasma IAPP (pM)	241.11±20.21	252.65±30.43
Total IgA (mg/ml)	46.74±51.31	84.23±47.65

[a]Data is presented for illustrative purposes and was not analyzed. Data was analyzed using either Student's t-test or Mann-Whitney test, and values are presented as mean value ± SD. Aβ - amyloid beta, APOE – apolipoprotein E, IAPP – islet amyloid polypeptide, Ig – immunoglobulin, PMD – postmortem delay, T2D – Type 2 diabetes.

Table S9. Demographic data and neuropathological assessment of individuals included in Cohort II.

Clinical diagnosis	Age (years)	Sex (M/F)	*APOE* genotype	T2D (-/+)	Aβ/NFT/LB[a]	Cause of death
AD	90	F	2/3	-	B/2/5	General physical deterioration, palliative sedation
AD	90	F	3/3	-	C/6/5	General deterioration in end-stage dementia syndrome
AD	83	M	3/4	-	B/3/0	Dehydration after CVA and mixed vascular/Alzheimer's dementia
AD	64	F	4/4	-	C/4/0	Euthanasia with progressed AD
AD	72	M	3/4	-	C/6/0	Pneumonia and stomach bleeding
AD	76	M	4/4	-	C/6/0	Dehydration, pneumonia
AD	91	F	3/4	-	C/5/5	Palliative care after CVA
AD	64	M	3/4	-	C/6/5	Euthanasia
AD	74	M	3/4	+	C/4/6	Dehydration
AD	83	M	3/3	-	C/4/6	Gastroenteritis or respiratory tract infection by advanced dementia syndrome
AD	88	F	3/3	-	C/5/5	Cachexia with vascular dementia
AD	91	F	3/3	+	C/4/0	CVA
AD	88	F	3/4	-	C/5/0	Pneumonia/palliative sedation
AD	65	F	3/4	-	C/5/1	Dehydration by respiratory tract infection by end-stage dementia
AD	92	F	3/4	-	C/6/0	3rd degree atrioventricular block and severe AD
AD	63	M	4/4	+	C/4/6	Hepatic insufficiency on the bases of metastases from unknown tumor
FTD	69	F	3/3	+	O/0/0	Infection
HA	72	M	3/4	-	O/2/0	Pneumonia
MS	78	M	3/3	+	O/2/0	Euthanasia
MS	87	F	3/3	+	A/2/0	Dehydration and renal insufficiency
MS	60	F	3/4	+	A/0/0	MS with decreased intake
NC	75	F	3/3	-	O/1/0	Euthanasia
NC	92	F	3/3	-	A/1/3	Euthanasia
NC	87	F	3/3	-	B/1/4	Respiratory insufficiency by pneumonia and exhaustion
NC	75	M	3/3	-	A/1/0	Cardiac arrest with COPD
NC	70	M	2/3	-	O/1/3	Pneumonia with cardiogenic shock
NC	68	F	3/3	+	O/1/0	Euthanasia
NC	81	M	3/4	-	C/3/0	Terminal pancreas carcinoma
VaD	93	F	3/3	-	B/2/0	Heart failure

[a]ABC staging of amyloid beta (Aβ), Braak staging of neurofibrillary tangles (NFT) and Lewy bodies (LB). Aβ - amyloid beta, AD – Alzheimer's disease, APOE – apolipoprotein E, COPD – chronic obstructive pulmonary disease, CVA – cerebrovascular accident, F – female, LB – Lewy body, FTD – frontotemporal dementia, HA – hippocampal alterations, M – male, MS – multiple sclerosis, NC – non-demented control, NFT – neurofibrillary tangle, T2D – Type 2 diabetes, VaD – vascular dementia.

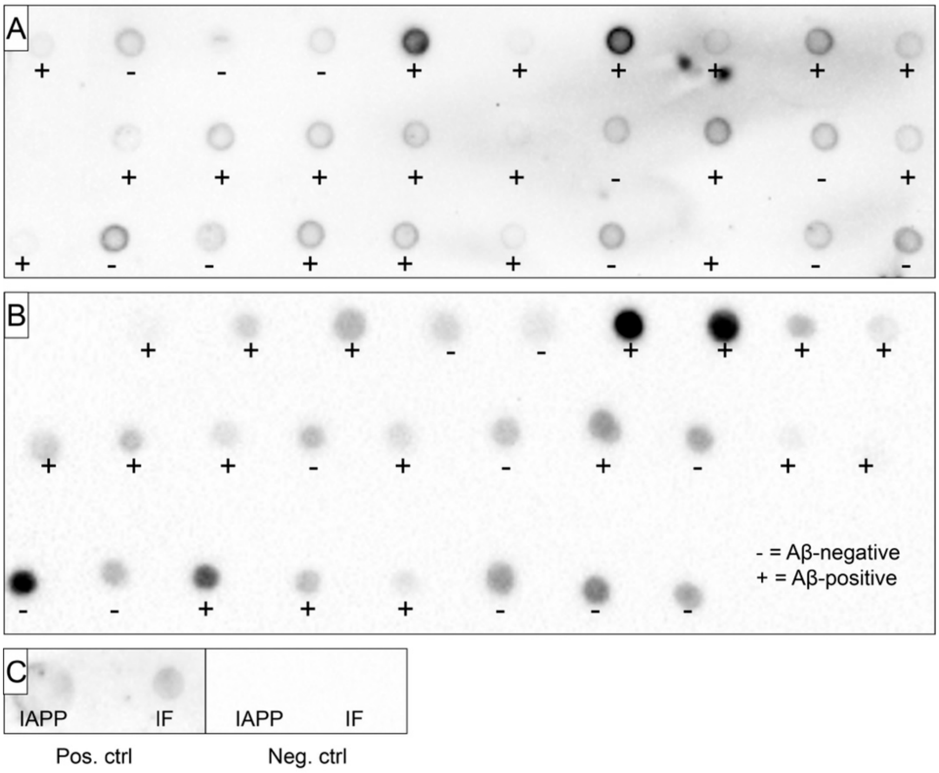

Figure S1. Dot-blot of brain insoluble and soluble IAPP fractions. The dot-blot shows brain insoluble (A) and soluble (B) IAPP fractions in samples from Aβ-negative (-) and Aβ-positive (+) cases. Positive and negative controls, an IAPP peptide (EZHA-52K, Merck, Sweden) on the left and a sample with IAPP-IF on the right, are indicated in (C).

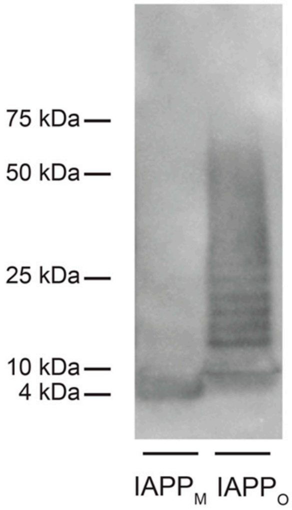

Figure S2. Characterization of IAPP self-assembly products. Western-Blot analysis of IAPP monomers ($IAPP_M$) and oligomers ($IAPP_O$).
